# Exploring Medical Student Experiences of the Use of Escape Rooms as an Innovative Teaching Method Within Undergraduate Psychiatry Teaching

**DOI:** 10.1192/bjo.2025.10287

**Published:** 2025-06-20

**Authors:** Hanna Mansi, Rachel Rice, Lisa Dikomitis, Sukhi Shergill, Joanne Rodda

**Affiliations:** 1 KMPT, Maidstone, United Kingdom; 2 Kent and Medway Medical School, Canterbury, United Kingdom; 3 University of Kent, Canterbury, United Kingdom; 4 KCL, London, United Kingdom

## Abstract

**Aims:** The study aimed to explore the experience and opinions of third year medical students regarding the use of escape rooms as a teaching modality in undergraduate psychiatry education. This research will help inform whether escape rooms have a viable role in medical education.

**Methods:** This was a mixed method study using quantitative and qualitative elements. Quantitative data was captured on Likert scales, through survey completion, immediately after completion of an escape room (n=64) based on their psychiatry curriculum at a revision day. The qualitative element of study included two focus groups of students (n=7), who had attended the revision day, to discuss their experience of escape rooms in medical education. Focus groups were audio-recorded and transcribed. The transcripts were then analysed using thematic analysis.

**Results:** 97% of surveyed students found the escape room an enjoyable experience. 57 (89%) students reported a positive improvement in knowledge, with only one student disagreeing. 72% of students reported it had improved their clinical skills. Free text responses included ‘really good revision exercise’ and ‘made revision good fun’. The main themes identified from focus groups were that escape rooms were an interactive and enjoyable learning experience which improved knowledge and allowed students to identify knowledge gaps to focus their revision. They enjoyed working in smaller groups, which they found inclusive, and some students felt this improved their team working skills. Some students found that the timed and competitive element detracted from the educational aspect and negatively impacted team working skills, whilst others valued this.

**Conclusion:** The findings of this study suggest that escape rooms could provide an innovative approach to teaching methods in undergraduate psychiatry. In this study, students found the escape room an enjoyable and beneficial experience that improved their knowledge, and they perceived a benefit for revision as the day was situated close to final year examinations. Equally, escape rooms could be used in a myriad of other ways to support learning in different contexts. Given the popularity of experiential learning in medical education this may be an option to provide positive experiences in undergraduate psychiatric education as well as meaningful educational experiences.

